# Novel groups and unique distribution of phage *phoH* genes in paddy waters in northeast China

**DOI:** 10.1038/srep38428

**Published:** 2016-12-02

**Authors:** Xinzhen Wang, Junjie Liu, Zhenhua Yu, Jian Jin, Xiaobing Liu, Guanghua Wang

**Affiliations:** 1 Key Laboratory of Mollisols Agroecology, Northeast Institute of Geography and Agroecology, Chinese Academy of Sciences, Harbin 150081, China; 2 University of Chinese Academy of Sciences, Beijing 100049, China

## Abstract

Although bacteriophages are ubiquitous in various environments, their genetic diversity is primarily investigated in pelagic marine environments. Corresponding studies in terrestrial environments are few. In this study, we conducted the first survey of phage diversity in the paddy ecosystem by targeting a new viral biomarker gene, *phoH*. A total of 424 *phoH* sequences were obtained from four paddy waters generated from a pot experiment with different soils collected from open paddy fields in northeast China. The majority of *phoH* sequences in paddy waters were novel, with the highest identity of ≤70% with known *phoH* sequences. Four unique groups (Group α, Group β, Group γ and Group δ) and seven new subgroups (Group 2b, Group 3d, Group 3e, Group 6a, Group 6b, Group 6c and Group 6d) were formed exclusively with the clones from the paddy waters, suggesting novel phage *phoH* groups exist in the paddy ecosystem. Additionally, the distribution proportions of *phoH* clones in different groups varied among paddy water samples, suggesting the phage community in paddy fields is biogeographically distributed. Furthermore, non-metric multidimensional scaling analysis indicated that phage *phoH* assemblages in paddy waters were distinct from those in marine waters.

The paddy field is a unique agro-ecosystem in which flooding and drainage are repeated during the annual cycle of rice cultivation, which results in the alternation of aerobic and anoxic processes in the paddy field ecosystem. Thus, the paddy field ecosystem is considered to be a hotspot for studying microbial ecology and biochemical cycles^1^,^2^. A large body of literature addresses the microbial ecology of paddy fields, including total bacterial and fungal communities^3^, methanogenic archaea^4^, methanotrophic bacteria^5^, and ammonia-oxidizing bacteria and archaea^6^. Recently, research on viral ecology or phage ecology in paddy ecosystems has aroused much attention^7^,^8^,^9^,^10^,^11^,^12^,^13^,^14^,^15^. For instance, Nakayama *et al*.^9^ observed that the abundance of virus-like particles (VLPs) in paddy floodwaters during the rice cultivation period ranged from 5.6 × 10^6^ to 1.2 × 10^9^ VLPs·mL^−1^ with a mean of 1.5 × 10^8^ VLPs·mL^−1^, which was greater than the number of viruses in oceans and estuaries^16^,^17^. Chae *et al*.^18^ isolated 34 phages infecting *Xanthomonas oryzae* pv. *oryzae* from paddy floodwaters and observed that using a phage mixture is an effective method to control the occurrence of rice bacterial leaf blight disease. Moreover, many novel phage sequences or specific phage groups were observed in paddy fields by analysing several biomarker genes^12^,^13^,^19^,^20^,^21^,^22^.

*The phoH* gene is a host-derived auxiliary metabolic gene (AMG) carried by some phages^23^. This gene belongs to the Pho regulon and regulates phosphate uptake and metabolism under conditions of low-phosphate and phosphate limitation^24^,^25^. Unlike popular biomarker genes (*g23, g20*, DNA *pol* and *psbA)*, that are restricted to the analysis of the genetic diversity of specific morphological phages, *phoH* is carried by various morphological types of phages (including siphophages, myophages and podophages), phages having wide host range (including autotrophic hosts and heterotrophic hosts), and even viruses of autotrophic eukaryotes^23^. By targeting this gene, Goldsmith *et al*.^23^ designed the degenerate primers vPhoHf/vPhoHr and were the first to use *phoH* to examine marine phage diversity throughout a depth profile in the Sargasso Sea and worldwide oceans. They found that viral *phoH* sequences in marine waters were highly diverse, and they identified six novel groups of *phoH* sequences. Subsequently, they further investigated the viral community composition throughout the water column both in summer and in winter across three years at the Bermuda Atlantic Time-series Study site in the Sargasso Sea, and this study revealed that the distribution patterns of viral communities varied not only with depth but also with time^26^,^27^. Their findings indicated that *phoH* is an effective signature gene for examining phage diversity in marine environments.

In researching the genetic diversity of phages in paddy ecosystems, we have previously found that several degenerate primers used for investigating phage diversity in marine environments, such as MZIAbis/MZIA6^28^, CPS1/CPS8^29^, psbA-F/psbA-R^30^ and CP-DNAP-349F/CP-DNAP-533Ra/b^31^, were also suitable for studying phage diversity in paddy ecosystems. Our overall findings showed that the phage communities were significantly different between paddy and marine ecosystems. In this study, to further understand the phage communities in paddy ecosystems, we targeted the *phoH* gene by using the primers vPhoHf/vPhoHr with the goal of addressing the following questions: (i) Do phages carry the *phoH* gene in paddy ecosystems? (ii) If so, how diverse and novel are they compared with reported sequences? (iii) Are the phage community compositions similar or different among different paddy fields or between paddy and marine ecosystems?

## Materials and Methods

### Sample collection and processing

An incubation experiment was designed to survey phage *phoH* genes in paddy waters in northeast (NE) China. The reason for using an incubation experiment rather than sampling floodwater from open paddy fields was to ensure that the phages were actually generated from the paddy fields. Because paddy fields in NE China are occasionally irrigated with river water or underground water, inappropriate sampling times directly from the open fields might result in data that do not truly reflect phages normally present in paddy waters. In brief, approximately 20 kg of soil (0~10 cm depth) were collected from the paddy fields of Daan (45°36′N, 123°50′E), Suihua (46°43′N, 126°59′E), Mudanjiang (44°26′N, 129°29′E), and Yanjiagang (45°35′N, 126°20′E) (Table S1) in NE China on 9~13 May, 2014. Each paddy soil sample was subpackaged equally into two plastic containers with dimensions of 60 × 40 × 28 cm and incubated with autoclaved water. One week later, after basal nutrients of 0.4 g KCl, 1.0 g Ca_3_(PO_4_)_2_, 1.0 g (NH_4_)_2_SO_4_ per kilogram of soil were added to the soil for rice growth, we transplanted eleven rice seedlings (*Oryza sativa* L. ssp. *japonica, cv*. Daohuaxiang) into each container on 22 May, 2014. Hereafter, all the plastic containers were put outside on days without rain and in the greenhouse on rainy days, and the water layer was maintained 8 cm above soil by timely supplementation with autoclaved water.

Approximately 1 L of water samples from the two containers of each soil sample was collected on 22 June. After being centrifuged twice at 5,000 rpm for 30 min at 4 °C to remove soil particles, plankton and partial bacteria, the supernatant was sequentially filtered through 0.4-μm and 0.2-μm carbonate membrane filters (Nuclepore Track-Etched Membranes, Whatman International Ltd, London, UK) to completely remove bacterial cells. Finally, virus-sized particles were filtered onto 0.03-μm carbonate membrane filters (Nuclepore Track-Etched Membranes, Whatman International Ltd, London, UK). The filters were placed in sterilized 2 mL centrifuge tubes and kept at −20 °C.

### DNA extraction and PCR amplification

The 0.03-μm filters were treated with DNase and RNase (40 μg mL^−1^ each) in 10 mM Tris-HCl buffer (pH 7.5) to decompose free DNA and RNA. Next, viral DNA extraction was performed according to the protocol reported previously^12^. The extracted DNA was dissolved in 30 μL TE buffer (10 mM Tris-HCl, 1 mM EDTA, pH 8.0) and stored at −20 °C for further analysis.

The *phoH* sequences were amplified with the degenerate primers vPhoHf and vPhoHr^23^. PCR reactions were performed in a 50 μL mixture containing 10 μL EasyTaq buffer (TransGen Biotech, Beijing, China), 5 μL dNTPs (2.5 mM each; TransGen Biotech, Beijing, China), 0.5 μL forward and reverse primers (50 pmol each), 1.5 μL DNA template and 2 μL of Easy Taq DNA polymerase (TransGen Biotech, Beijing, China). The reactions were filled to the required volume with sterile Milli-Q water. The negative control contained all reagents and sterile Milli-Q water without the template. The thermal program used for PCR amplification was the same as a paper reported previously^23^.

### Cloning and sequencing

PCR products of approximately 420 bp in length were cut out from a 2% agarose gel and purified using the QIAExII Gel Extraction Kit (QIAGEN, Shanghai, China, Cat. No. 20021). The purified DNA was cloned into the pMD18-T vector (TaKaRa, Dalian, China) and transformed into competent cells of *Escherichia coli* DH5α. White clones were picked out for PCR amplification using the same primers and PCR program described above. After being harvested by overnight culture, the plasmid DNA from positive clones was sequenced by a commercial company (BGI, Shenzhen, China). The *phoH* nucleotide sequences obtained in this study were deposited in GenBank under accession numbers KX189635-KX190058.

### Phylogenetic analysis

The *phoH* sequences were translated to deduced amino acid sequences by the EMBOSS Transeq program on the European Bioinformatics Institute website (http://www.ebi.ac.uk/Tools/st/emboss_transeq/). The closest relatives of *phoH* clones were examined at the amino acid level using the BLASTp search program on the NCBI website (http://www.ncbi.nlm.nih.gov/BLAST). Reference *phoH* sequences from cultured viruses and bacteria, as well as environmental viral clones were retrieved from GenBank. After the amino acid sequences were aligned with CLUSTALX 1.81^32^, neighbour-joining phylogenetic trees were constructed using software (MEGA 4.0)^33^ with 1,000 bootstrap replicates.

### Clone library and diversity analyses

Operational taxonomic units (OTUs) were generated based on sequence similarity of 97% at the nucleotide level. Non-metric multidimensional scaling (NMDS) analysis was used to visually display the differences of phage *phoH* sequence assemblages based on the distances between clone libraries. The NMDS analysis was performed in R-2.15.1^34^ with the “vegan” package^35^. Accession numbers for the reference sequences used in this analysis are shown in Table S2.

## Results

### Closest relatives of *phoH* clones

Out of 486 positive clones submitted for sequencing, 424 clones were identified as *phoH* sequences. Among these sequences, 166, 97, 36 and 125 clones were from water samples of Daan, Suihua, Mudanjiang, and Yanjiagang, respectively. The *phoH* fragments had lengths of 381~405 bp, encoding 127~135 amino acid residues (Table S3). The BLASTp search for the closest relatives at the amino acid level showed that 108, 114, 40 and 4 clones had the highest identity to viral *phoH* clones from the Sargasso Sea, the Mediterranean Sea, the Gulf of Mexico and British Columbia coastal waters, respectively^23^. Additionally, 75 clones had the highest identity to *Synechococcus* phage S-SSM7 from the Sargasso Sea^36^, 31 clones had the highest identity to an uncultured phage uvMED from the Mediterranean Sea, Spain^37^, 20 clones had the highest identity to *Synechococcus* phage S-RSM4 from the Red Sea^38^, and 32 clones had the highest identity to *Acinetobacter* phage YMC13/03/R2096 from South Korea (Table S3; Table S4). Noticeably, approximately 70% (295/424) of the clones had identity ≤70% with known *phoH* sequences, which indicated that the majority of *phoH* clones obtained in this study are novel (Table S3; Table S4).

### Phylogeny of *phoH* sequences

**Figure 1:**
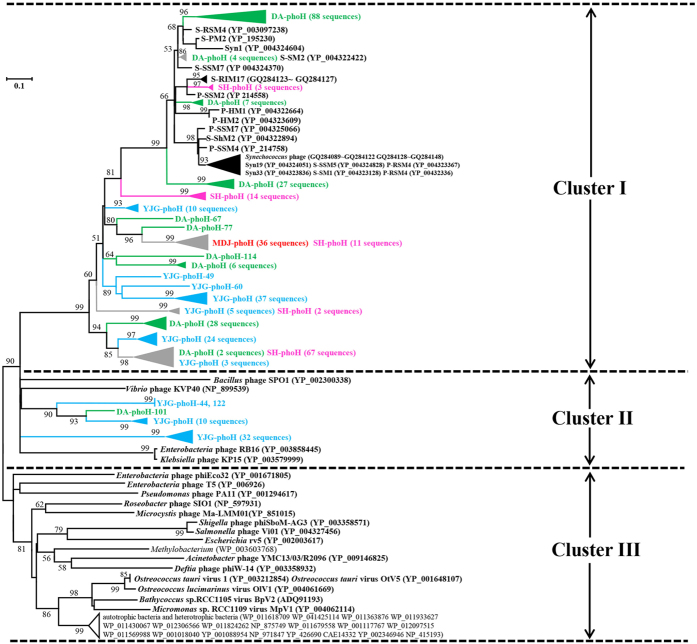
Neighbour-joining phylogenetic tree of *phoH* amino acid sequences obtained in this study with reference *phoH* sequences of cultured phages of autotrophs and heterotrophs, viruses of eukaryotic autotrophs, and cultured autotrophic and heterotrophic bacterial hosts. Clones in this study were named to reflect the sampling site, biomarker gene name and clone number. In detail, DA, SH, MDJ and YJG indicated samples from Daan (green words in bold), Suihua (pink words in bold), Mudanjiang (red words in bold) and Yanjiagang (blue words in bold), respectively. The green, pink and blue triangles indicate *phoH* clusters obtained from Daan, Suihua and Yanjiagang, respectively, and the grey triangles represent *phoH* clusters obtained from more than one paddy sample. The *phoH* sequences of isolated virus and phage origins are in bold black font, and their clusters are indicated with black triangles. The *phoH* sequences of isolated host origins are in normal black font, and their clusters are indicated with white triangles. Bootstrap values <50 are not shown. The scale bar represents the abundance of amino acid substitutions per residue.

All the *phoH* clones obtained in this study were used to build a phylogenetic tree with *phoH* sequences from cultured phages of autotrophs and heterotrophs, and *phoH* sequences from cultured autotrophic and heterotrophic bacterial hosts (Fig. 1). Overall, the phylogenetic tree could be mainly divided into three clusters (Clusters I, II and III). Cluster I contained phages infecting autotrophic bacteria, Cluster II contained phages infecting heterotrophic bacteria, and Cluster III contained viruses infecting eukaryotes, phages infecting several heterotrophic bacteria, one phage of an autotrophic bacterium (*Microcystis* phage Ma-LMM01), and numerous host *phoH* genes from heterotrophic and autotrophic bacteria. It should be noted that approximately 90% (379/424) of *phoH* clones observed in this study were grouped into Cluster I (phages of autotrophic bacteria), while the remaining 45 clones were grouped into Cluster II (phages of heterotrophic bacteria). No clones from this study were grouped into Cluster III.

**Figure 2:**
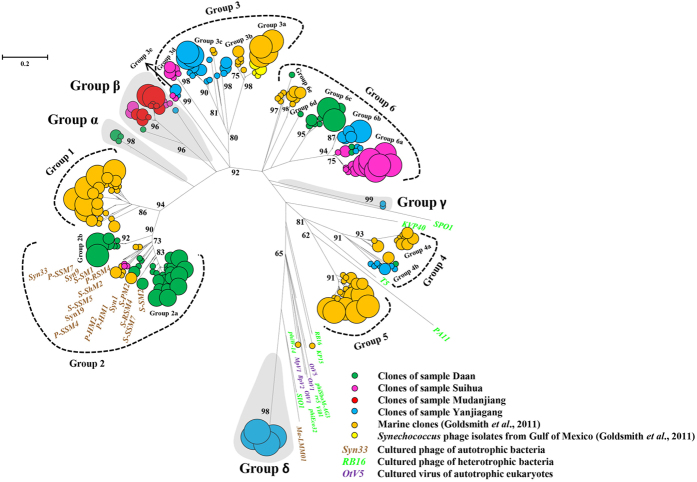
Unrooted phylogenetic tree comparing *phoH* amino acid sequences of environmental clones obtained from this study and marine waters, cultured phages of autotrophic and heterotrophic bacteria, as well as cultured eukaryotic viruses. The bootstrap values <50 are not shown. The size of circles at the end of branches is proportional to the number of clones/phages, and the small, medium and large circles represent one, four and eight clones/phages, respectively. The scale bar represents the abundance of amino acid substitutions per residue.

To determine whether novel phage *phoH* groups exist in paddy waters, the phylogenetic relationships of all sequences observed in this study with *phoH* sequences coming from marine water clones, *Synechococcus* phage isolates, cultured phage of autotrophic and heterotrophic bacteria, and cultured viruses of autotrophic eukaryotes at the amino acid level are shown in Fig. 2. According the grouping standard first designed by Goldsmith *et al*.^23^, the viral *phoH* sequences from six worldwide oceans were phylogenetically distributed into Groups 1, 2, 3, 4, 5 and 6, of which Group 3 was further divided into Group 3a, 3b and 3c. In this study, we found that 30.42%, 16.27%, 2.60% and 29.72% of clones across all samples were grouped into the previously designed Groups 2, 3, 4 and 6, respectively. No clones were grouped into Groups 1 and 5. In addition, 6, 49, 2 and 32 clones from this study formed four new groups, named as Group α, Group β, Group γ, and Group δ, respectively. No clones from marine waters were grouped into the four newly designed groups. Furthermore, within formerly named groups, seven new subgroups, *i.e*., Group 2b, Group 3d, Group 3e, Group 6a, Group 6b, Group 6c and Group 6d, were formed exclusively with the clones obtained in this study.

### Biogeography of phage *phoH* sequences

The distribution proportions of *phoH* clones into different groups not only differed between marine waters and paddy waters but also differed among paddy water samples (Table 1; Fig. S1). For example, over 60% (99/166) of clones from Daan and 3% of clones from Suihua (but no clones from other two water samples) were grouped into Group 2a. Approximately 69% of clones from Suihua were grouped into Group 6a, while less than 3% of clones from other water samples were grouped into that group. Approximately 26%, 38% and 19% of clones from Yanjiagang were grouped into Group δ, Group 3c, and Group 6b, respectively, and no clones from other samples were grouped into those groups. All clones from Mudanjiang were grouped into Group β, while only about 11% of clones from Suihua and less than 1% of clones from both Daan and Yanjiagang were grouped into Group β.

**Figure 3:**
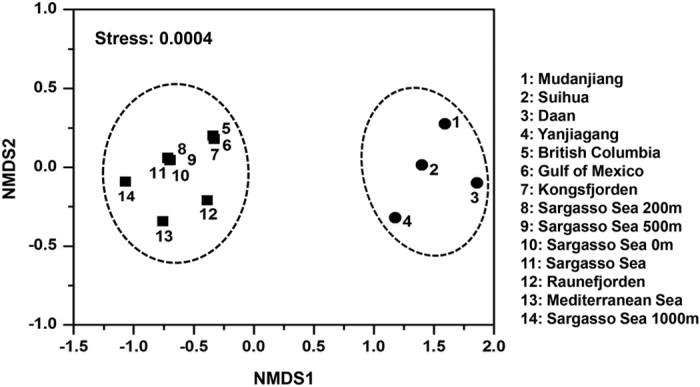
Non-metric multidimensional scaling analysis of phage *phoH* communities. The plot shows the distribution pattern of the phage *phoH* sequence assemblages obtained from different environments. Samples located in close proximity on the NMDS plot are grouped by dashed circles.

To show clearly the distribution patterns of phage communities in different environments, the phage *phoH* sequence assemblages from paddy waters of this study and from marine environments^23^ including the Sargasso Sea, the Mediterranean Sea, the Gulf of Mexico, British Columbia coastal waters, Raunefjorden and Kongsfjorden were subjected to NMDS analysis (Table S2). The plot clearly showed that all samples were separated into two groups (Fig. 3). One group contained samples from marine waters, and another group included samples from paddy waters. This finding indicated that phage *phoH* assemblages in paddy waters were distinct from those in marine waters.

## Discussion

### PCR amplification of the *phoH* genes in paddy waters

The primer set vPhoHf/vPhoHr was originally designed by Goldsmith *et al*.^23^ to amplify the phage *phoH* genes from marine environments. They found that the *phoH* gene is commonly carried in phages that infect heterotrophic and autotrophic bacteria, as well as in viruses infecting autotrophic eukaryotes. Unlike other biomarker genes, such as *g23, g20, psbA* and DNA *pol*, which only target a specific family of phages, the *phoH* gene is not restricted to a certain morphological type of phage, which suggests that it could be a powerful biomarker gene for studying phage diversity. Moreover, by comparing the fully sequenced phage genomes in GenBank, they found that nearly 40% of marine phages contained the *phoH* gene, while only 4% of nonmarine phages contained this gene. This finding seemed to restrict the application of this gene to study phage diversity in terrestrial environments. However, only a small fraction of bacteria can be cultured^39^,^40^, which may hamper our ability to isolate cultured phages. Thus, many viruses in natural environments that might contain this gene may not have been identified due to current culture conditions. In this study, we pioneered the use of this gene to survey the diversity of phages in paddy waters. We collected the viral particles, ultimately excluded contamination from hosts or host’s DNA, and obtained 424 *phoH* clones from four paddy water samples. We found all clones were phylogenetically grouped into Cluster I and Cluster II (Fig. 1), and Clusters I and II contained several *phoH* references coming from phages of autotrophic and heterotrophic bacteria, respectively. None of the *phoH* sequences from viruses of eukaryotes and hosts were grouped into these two clusters (Fig. 1). This finding strongly demonstrated that the *phoH* gene was contained in phage genomes of terrestrial environments and that this biomarker gene was useful for studying phage ecology in paddy ecosystems.

### Phylogenetic position of *phoH* genes in paddy waters

The pioneering work of Goldsmith *et al*.^23^ showed that phage-originated *phoH* sequences could be separated from host *phoH* sequences through constructing a phylogenetic tree, and they also stated that autotrophic phages and heterotrophic phages tended to cluster separately. In this study, we found that nearly 90% of *phoH* clones formed a well-supported (99%) cluster in Cluster I with several cyanophages infecting *Synechococcus* and *Prochlorococcus*. No *phoH* sequences from cultured phages of heterotrophic bacteria, viruses of eukaryotes, or hosts were grouped into Cluster I (Fig. 1). This suggested that Cluster I clones might originate from cyanophages from paddy water. Many clades in Cluster I of Fig. 1 have no reference sequences, which might be due to great diversity of cyanobacteria in the paddy field. Consistent with that speculation, our previous study has already revealed that many unknown groups of picocyanobacteria exist in paddy fields^22^. In contrast, less than 10% of clones in this study (mainly obtained from Yanjiagang) formed two clades in Cluster II closely related to phages infecting heterotrophic bacteria, suggesting that those clones might be from noncyanophages (Fig. 1). This heavily disproportionate split of *phoH* clones, between those that group with cyanophages and those that group with noncyanophages, may imply that in paddy waters, this auxiliary metabolic gene is mainly contained in genomes of cyanophages. Future studies using culture-dependent methods are needed to address this implication.

Goldsmith *et al*.^23^ obtained 289 phage *phoH* sequences from the Sargasso Sea, British Columbia coastal waters, the Gulf of Mexico, Raunefjorden, Kongsfjorden and the Mediterranean Sea. They found that the majority of those sequences were grouped into six groups, and they labeled those groups as Groups 1–6. Further sequencing by a high throughput method revealed no new phylogenetic groups in the Sargasso Sea^26^. In this study, we obtained 424 phage *phoH* clones from paddy waters. Among them, approximately 80% of clones were grouped into Groups 2, 3, 4 and 6, while 20% of clones formed four new groups (Group α, Group β, Group γ and Group δ). In addition, several new subgroups within individual groups were formed exclusively with the clones obtained from paddy waters (Fig. 2). These findings suggest that the distribution pattern of phage *phoH* genes in paddy waters is somewhat distinct from that found in marine waters.

Comparing the distribution patterns between Figs 1 and 2, we found that all clones obtained in this study in Cluster I of Fig. 1 were located in Groups 2, 3, 6, α and β, while the clones in Cluster II of Fig. 1 were distributed into Group 4, γ and δ (Fig. 2). As elaborated above, we speculated the sources of *phoH* sequences obtained in paddy waters in Groups 2, 3, 6, α and β may be cyanophages. This speculation was bolstered by the phylogenetic tree of Fig. 2, in which Groups 1, 2, 3, α and β formed a large well-supported cluster (92%) containing several reference *phoH* sequences of cyanophages^23^,^26^ (Fig. 2). Although no known phage *phoH* sequences were grouped into Group 6, all the marine *phoH* sequences in Group 6 were obtained from surface and upper water samples, and no clones from 1000 m depth of the Sargasso Sea (where cyanophages were absent) were grouped into this group^23^. In addition, all the paddy *phoH* clones that fell into Group 6 (Fig. 2) were located in Cluster I of Fig. 1. Together, these findings strongly implied that the *phoH* clones in Group 6 might also originated from cyanophages. Future research is needed to address this possibility.

### Biogeographical distribution of phage *phoH* sequences in paddy waters

Previous studies showed that the composition of viral *phoH* sequences not only varied throughout the water column but also changed throughout the year in the Sargasso Sea^23^,^26^,^27^. In addition, the composition of viral *phoH* sequences was also different among six worldwide oceans^23^. Those findings indicated that the composition of viral *phoH* sequences in oceans was spatially and temporally distributed. In this study, although the paddy water samples were collected only one time for surveying the diversity of viral *phoH* genes, we found that the distribution proportions of *phoH* clones into different groups varied among paddy water samples (Table 1; Fig. S1), which suggested that the phage *phoH* genes in paddy fields were biogeographically distributed. Furthermore, comparison of the assemblages of viral *phoH* sequences showed that the viral communities were distinctly different between paddy waters and ocean waters (Fig. 3). Similar findings have also been observed by analysing other biomarker genes in paddy fields, such as *g*23^41^, *g*20^15^ and DNA *pol*^13^.

Although different *phoH* compositions were observed among paddy waters, and between paddy waters and marine waters, we still found that certain *phoH* groups were commonly detected in multiple samples, which suggested that some phages are not restricted by geographical separation. Sano *et al*.^42^ reported that the phages obtained from soil, freshwater and sediment can propagate on hosts from the marine environment. Moreover, they also showed that marine phages from one location can infect hosts from a different marine location. Others studies on phage ecology targeting different biomarker genes, such as *g23* of T4-type phages^43^, *g20* of cyanomyophages^44^ and DNA *pol* gene of podophages^45^, have also detected identical sequences across widely separated geographical locations and different habitats. Those findings suggested that some phages are not host-specific. It is a universal phenomenon in natural conditions that one host can be infected by different phages or a phage can infect different hosts^46^. This phenomenon promotes horizontal gene transfer between phages and hosts, and even across different hosts, which promotes evolution.

## Conclusions

In conclusion, 424 *phoH* clones were obtained from paddy waters. Among them, approximately 90% of the *phoH* clones were grouped with cyanophages, while 10% of the clones were grouped with phages of heterotrophic bacteria. This division implied that this auxiliary metabolic gene was carried mainly in the genomes of cyanophages in paddy waters. Four new groups and seven new subgroups were formed exclusively with the clones from paddy waters, suggesting that the distribution pattern of the phage *phoH* gene in paddy waters was distinct from that in marine waters. In addition, the phage community compositions represented by *phoH* sequences varied among paddy water samples and were also remarkably different from those observed in marine waters. As far as we know, this is the first study revealing that phages in paddy fields also contain the *phoH* gene, suggesting this biomarker gene is an effective signature gene for investigating phage diversity both in marine and terrestrial environments.

## Additional Information

**How to cite this article**: Wang, X. *et al*. Novel groups and unique distribution of phage *phoH* genes in paddy waters in northeast China. *Sci. Rep.* **6**, 38428; doi: 10.1038/srep38428 (2016).

**Publisher's note:** Springer Nature remains neutral with regard to jurisdictional claims in published maps and institutional affiliations.

## Supplementary Material

Supplementary Information

## Figures and Tables

**Table 1:** Number and distribution proportion of the phage *phoH* clones in phylogenetic groups obtained from marine waters and paddy waters.

Phylogenetic groups	Marine^a^ (275)^d^		Paddy^b^ (424)^d^		Daan^c^ (166)^d^		Suihua^c^ (97)^d^		Mudanjiang^c^ (36)^d^		Yanjiagang^c^ (125)^d^	
	Number of clones	Proportion (%)	Number of clones	Proportion (%)	Number of clones	Proportion (%)	Number of clones	Proportion (%)	Number of clones	Proportion (%)	Number of clones	Proportion (%)
Group α			6	1.42	6	3.62						
Group β			49	11.56	1	0.60	11	11.34	36	100	1	0.80
Group γ			2	0.47							2	1.60
Group δ			32	7.55							32	25.60
Group 1	91	33.09										
Group 2a	13	4.73	102	24.05	99	59.64	3	3.09				
Group 2b			27	6.37	27	16.27						
Group 3a	33	12										
Group 3b	8	2.91										
Group 3c	2	0.73	48	11.32							48	38.40
Group 3d			14	3.30			14	14.44				
Group 3e			7	1.65			2	2.06			5	4
Group 4a	4	1.45										
Group 4b	24	8.72	11	2.59	1	0.60					10	8
Group 5	77	28										
Group 6a			72	16.98	2	1.20	67	69.07			3	2.40
Group 6b			24	5.66							24	19.20
Group 6c			28	6.60	28	16.87						
Group 6d			1	0.24	1	0.60						
Group 6e	21	7.64	1	0.24	1	0.60						
Undesigned group	2	0.73										

^a^Marine sources including the Sargasso Sea and worldwide oceans (Goldsmith *et al*.^23^).

^b^Paddy sources including the four paddy sampling sites (Daan, Suihua, Mudanjiang and Yanjiagang) in this study.

^c^Paddy sample in this study.

^d^Number in parenthesis is the total number of clones obtained from each source/sample.
