# Supplementary material for: Novel groups and unique distribution of phage phoH genes in paddy waters in northeast China
Source: Sci Rep. 2016 Dec 2;6:38428. doi: 10.1038/srep38428 (PMC5133604; doi:10.1038/srep38428)
Supplement: Supplementary Information [file srep38428-s1.pdf]

Title: Novel groups and unique distribution of phage *phoH* genes in paddy waters in northeast China

Author names: Xinzhen Wang[1,2], Junjie Liu[1], Zhenhua Yu[1], Jian Jin[1], Xiaobing Liu[1], Guanghua Wang[1,*]

Affiliation:

[1]*Key Laboratory of Mollisols Agroecology, Northeast Institute of Geography and Agroecology, Chinese Academy of Sciences, Harbin 150081, China*

[2]*University of Chinese Academy of Sciences, Beijing 100049, China*

[*]**Corresponding author:** Guanghua Wang

Corresponding address: Key Laboratory of Mollisols Agroecology, Northeast Institute of Geography and Agroecology, Chinese Academy of Sciences, Harbin 150081, China

Tel: +86-451-86602745 **Fax:** +86-451-86603736

E-mail address: wanggh@iga.ac.cn; guanghuawang@hotmail.com

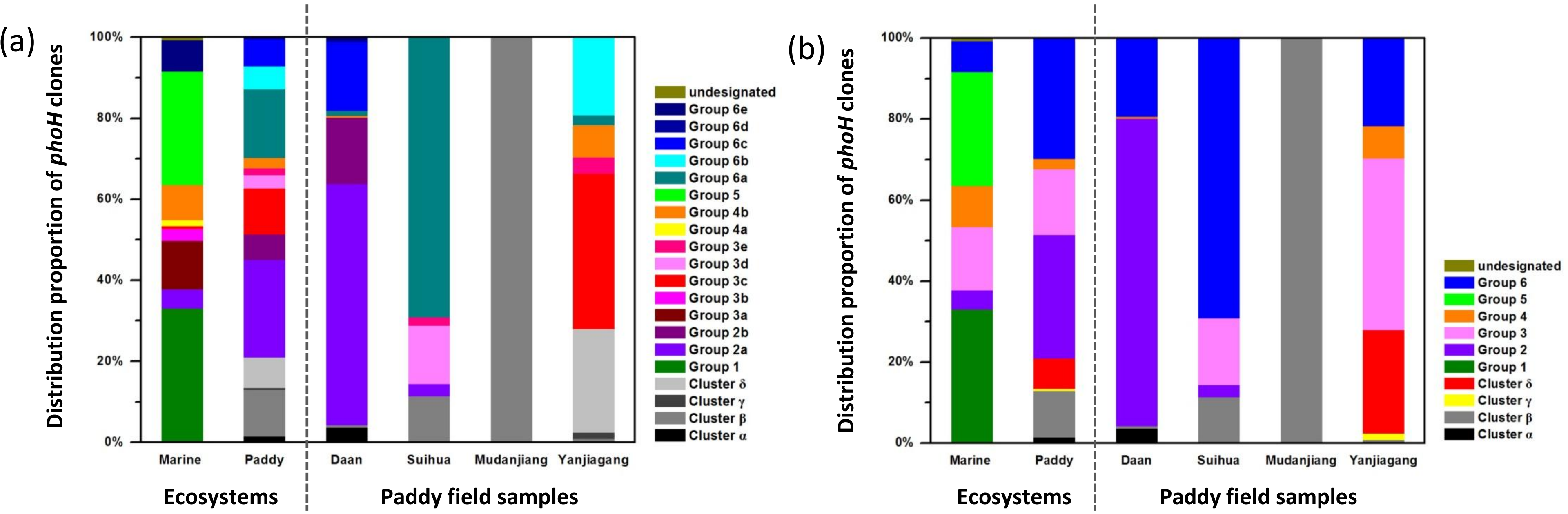

Fig. S1. Comparison of distribution proportions of the phage *phoH* clones in different groups. (a) The group/subgroup composition of each sample; (b) The overall group composition of each sample by collapsing the subgroups. The phage *phoH* clones were obtained from paddy samples from Daan, Suihua, Mudanjiang and Yanjiagang in this study and from marine samples from the Sargasso Sea and worldwide oceans[23].

Table S1. Properties of soil samples collected from open paddy fields.

Sample	Location	Soil type	Latitude and longitude	Total P (g kg^{-1})	Total C (g kg^{-1})	Total N (g kg^{-1})	C/N	pH
DA	Daan, Jilin	Saline-alkaline soil	45°36′N,123°50′E	0.45	12.52	0.47	26.64	7.46
SH	Suihua, Heilongjiang	Black soil	46°43′N,126°59′E	1.00	34.69	2.56	13.55	5.76
MDJ	Mudanjiang, Heilongjiang	Dark brown soil	44°26′N,129°29′E	0.63	15.21	0.99	15.36	6.10
YJG	Yanjiagang, Heilongjiang	Black soil	45°35′N,126°20′E	0.50	14.06	0.91	15.45	5.67

C, carbon; N, nitrogen; P, phosphorus.

Table S2. Source of phage *phoH* sequences used for the NMDS analysis.

Source	Sample site	NCBI accession number	Number of clones	Reference
Paddy	Daan	KX189635-KX190058	166	This study
	Suihua		97	
	Mudanjiang		36	
	Yanjiagang		125	
Marine	Raunefjorden	JF963974-JF964153<br>JF964160-JF964251	18	Goldsmith *et al*. 2011
	British Columbia		18	
	Gulf of Mexico		19	
	Kongsfjorden		19	
	Mediterranean Sea		18	
	Sargasso Sea		69	
	Sargasso Sea 0m		38	
	Sargasso Sea 200m		21	
	Sargasso Sea 500m		24	
	Sargasso Sea 1000m		28	

Table S3. The closest relative and the distribution of phylogenetic groups of sequenced *phoH* clones from the four paddy water samples at the amino acid level in this study.

Clone name	Length[a]	Closest relative			Alignment	Identity	Phylogenetic
		Clone/isolate	Source	Accession number[b]		(%)	group
SH-phoH-1	128	GOM_phoH_09	Gulf of Mexico	AEQ27527	70/127	55	Group β
SH-phoH-2	129	MED_phoH_13	Mediterranean surface water	AEQ27550	76/128	59	Group 6a
SH-phoH-3	129	SAR_0m_phoH_30	Sargasso Sea	AEQ27325	74/128	58	Group 3e
SH-phoH-4	129	MED_phoH_13	Mediterranean surface water	AEQ27550	75/128	59	Group 6a
SH-phoH-5	129	MED_phoH_13	Mediterranean surface water	AEQ27550	76/128	59	Group 6a
SH-phoH-6	129	MED_phoH_13	Mediterranean surface water	AEQ27550	76/128	59	Group 6a
SH-phoH-7	129	MED_phoH_13	Mediterranean surface water	AEQ27550	75/128	59	Group 6a
SH-phoH-8	129	MED_phoH_13	Mediterranean surface water	AEQ27550	76/128	59	Group 6a
SH-phoH-9	128	GOM_phoH_09	Gulf of Mexico	AEQ27527	70/127	55	Group β
SH-phoH-10	129	MED_phoH_13	Mediterranean surface water	AEQ27550	76/128	59	Group 6a
SH-phoH-11	128	GOM_phoH_09	Gulf of Mexico	AEQ27527	70/127	55	Group β
SH-phoH-12	132	SAR_0m_phoH_29	Sargasso Sea	AEQ27324	75/134	56	Group 3d
SH-phoH-13	129	MED_phoH_13	Mediterranean surface water	AEQ27550	75/128	59	Group 6a
SH-phoH-14	128	GOM_phoH_09	Gulf of Mexico	AEQ27527	70/127	55	Group β
SH-phoH-15	129	MED_phoH_13	Mediterranean surface water	AEQ27550	76/128	59	Group 6a
SH-phoH-16	129	MED_phoH_13	Mediterranean surface water	AEQ27550	75/128	59	Group 6a
SH-phoH-17	132	SAR_1000m_phoH_11	Sargasso Sea	AEQ27389	74/134	55	Group 3d
SH-phoH-18	129	MED_phoH_13	Mediterranean surface water	AEQ27550	75/128	59	Group 6a
SH-phoH-19	128	SAR_phoH_Mar_2011_0m_04	Sargasso Sea	AKV56976	69/127	54	Group β
SH-phoH-20	129	MED_phoH_13	Mediterranean surface water	AEQ27550	74/128	58	Group 6a
SH-phoH-21	132	SAR_0m_phoH_29	Sargasso Sea	AEQ27324	75/134	56	Group 3d
SH-phoH-22	132	SAR_0m_phoH_29	Sargasso Sea	AEQ27324	75/134	56	Group 3d
SH-phoH-23	129	MED_phoH_13	Mediterranean surface water	AEQ27550	76/128	59	Group 6a
SH-phoH-24	135	SAR_phoH_Sept_2010_0m_09	Sargasso Sea	AKV56959	114/134	85	Group 2a
SH-phoH-25	129	MED_phoH_13	Mediterranean surface water	AEQ27550	75/128	59	Group 6a
SH-phoH-26	132	SAR_0m_phoH_29	Sargasso Sea	AEQ27324	75/134	56	Group 3d
SH-phoH-27	129	MED_phoH_13	Mediterranean surface water	AEQ27550	76/128	59	Group 6a
SH-phoH-28	129	MED_phoH_13	Mediterranean surface water	AEQ27550	76/128	59	Group 6a
SH-phoH-29	132	SAR_phoH_Sept_2011_100m_02	Sargasso Sea	AKV57024	75/134	56	Group 3d
SH-phoH-30	135	SAR_phoH_Sept_2010_0m_09	Sargasso Sea	AKV56959	113/134	84	Group 2a
SH-phoH-31	129	MED_phoH_13	Mediterranean surface water	AEQ27550	76/128	59	Group 6a
SH-phoH-32	129	MED_phoH_13	Mediterranean surface water	AEQ27550	77/128	60	Group 6a
SH-phoH-33	129	MED_phoH_13	Mediterranean surface water	AEQ27550	74/128	58	Group 6a
SH-phoH-34	129	MED_phoH_13	Mediterranean surface water	AEQ27550	75/128	59	Group 6a
SH-phoH-35	129	MED_phoH_13	Mediterranean surface water	AEQ27550	76/128	59	Group 6a
SH-phoH-36	129	MED_phoH_13	Mediterranean surface water	AEQ27550	76/128	59	Group 6a
SH-phoH-37	132	SAR_1000m_phoH_11	Sargasso Sea	AEQ27389	74/134	55	Group 3d
SH-phoH-38	128	GOM_phoH_09	Gulf of Mexico	AEQ27527	70/127	55	Group β
SH-phoH-39	128	GOM_phoH_09	Gulf of Mexico	AEQ27527	70/127	55	Group β
SH-phoH-40	132	SAR_phoH_Sept_2011_100m_02	Sargasso Sea	AKV57024	74/134	55	Group 3d
SH-phoH-41	129	MED_phoH_13	Mediterranean surface water	AEQ27550	73/128	57	Group 6a
SH-phoH-42	132	SAR_0m_phoH_29	Sargasso Sea	AEQ27324	75/134	56	Group 3d
SH-phoH-43	129	MED_phoH_13	Mediterranean surface water	AEQ27550	76/128	59	Group 6a
SH-phoH-44	129	MED_phoH_13	Mediterranean surface water	AEQ27550	75/128	59	Group 6a
SH-phoH-45	129	MED_phoH_13	Mediterranean surface water	AEQ27550	76/128	59	Group 6a
SH-phoH-46	132	SAR_1000m_phoH_11	Sargasso Sea	AEQ27389	74/134	55	Group 3d
SH-phoH-47	129	MED_phoH_13	Mediterranean surface water	AEQ27550	76/128	59	Group 6a
SH-phoH-48	129	MED_phoH_13	Mediterranean surface water	AEQ27550	76/128	59	Group 6a
SH-phoH-49	129	MED_phoH_13	Mediterranean surface water	AEQ27550	77/128	60	Group 6a
SH-phoH-50	132	SAR_1000m_phoH_11	Sargasso Sea	AEQ27389	72/134	54	Group 3d
SH-phoH-51	129	MED_phoH_13	Mediterranean surface water	AEQ27550	75/128	59	Group 6a
SH-phoH-52	129	MED_phoH_13	Mediterranean surface water	AEQ27550	76/128	59	Group 6a
SH-phoH-53	129	MED_phoH_13	Mediterranean surface water	AEQ27550	76/128	59	Group 6a
SH-phoH-54	129	MED_phoH_13	Mediterranean surface water	AEQ27550	77/128	60	Group 6a
SH-phoH-55	129	MED_phoH_13	Mediterranean surface water	AEQ27550	76/128	59	Group 6a
SH-phoH-56	128	GOM_phoH_09	Gulf of Mexico	AEQ27527	70/127	55	Group β
SH-phoH-57	128	GOM_phoH_09	Gulf of Mexico	AEQ27527	70/127	55	Group β
SH-phoH-58	129	SAR_0m_phoH_30	Sargasso Sea	AEQ27325	73/128	57	Group 3e
SH-phoH-59	129	MED_phoH_13	Mediterranean surface water	AEQ27550	76/128	59	Group 6a
SH-phoH-60	129	MED_phoH_13	Mediterranean surface water	AEQ27550	76/128	59	Group 6a
SH-phoH-61	132	SAR_0m_phoH_29	Sargasso Sea	AEQ27324	75/134	56	Group 3d
SH-phoH-62	129	MED_phoH_13	Mediterranean surface water	AEQ27550	75/128	59	Group 6a
SH-phoH-63	129	MED_phoH_13	Mediterranean surface water	AEQ27550	76/128	59	Group 6a
SH-phoH-64	129	MED_phoH_13	Mediterranean surface water	AEQ27550	76/128	59	Group 6a
SH-phoH-65	129	MED_phoH_13	Mediterranean surface water	AEQ27550	76/128	59	Group 6a
SH-phoH-66	129	MED_phoH_13	Mediterranean surface water	AEQ27550	77/128	60	Group 6a
SH-phoH-67	129	MED_phoH_13	Mediterranean surface water	AEQ27550	76/128	59	Group 6a
SH-phoH-68	129	MED_phoH_13	Mediterranean surface water	AEQ27550	75/128	59	Group 6a
SH-phoH-69	132	SAR_0m_phoH_29	Sargasso Sea	AEQ27324	74/134	55	Group 3d
SH-phoH-70	129	MED_phoH_13	Mediterranean surface water	AEQ27550	76/128	59	Group 6a
SH-phoH-71	129	MED_phoH_13	Mediterranean surface water	AEQ27550	76/128	59	Group 6a
SH-phoH-72	129	MED_phoH_13	Mediterranean surface water	AEQ27550	76/128	59	Group 6a
SH-phoH-73	129	MED_phoH_13	Mediterranean surface water	AEQ27550	75/128	59	Group 6a
SH-phoH-74	129	MED_phoH_13	Mediterranean surface water	AEQ27550	75/128	59	Group 6a
SH-phoH-75	129	MED_phoH_13	Mediterranean surface water	AEQ27550	76/128	59	Group 6a
SH-phoH-76	129	MED_phoH_13	Mediterranean surface water	AEQ27550	76/128	59	Group 6a

SH-phoH-77	129	MED_phoH_13	Mediterranean surface water	AEQ27550	75/128	59	Group 6a
SH-phoH-78	129	MED_phoH_13	Mediterranean surface water	AEQ27550	76/128	59	Group 6a
SH-phoH-79	132	SAR_0m_phoH_29	Sargasso Sea	AEQ27324	75/134	56	Group 3d
SH-phoH-80	129	MED_phoH_13	Mediterranean surface water	AEQ27550	76/128	59	Group 6a
SH-phoH-81	129	MED_phoH_13	Mediterranean surface water	AEQ27550	76/128	59	Group 6a
SH-phoH-82	135	SAR_phoH_Sept_2010_0m_09	Sargasso Sea	AKV56959	113/134	84	Group 2a
SH-phoH-83	129	MED_phoH_13	Mediterranean surface water	AEQ27550	76/128	59	Group 6a
SH-phoH-84	129	MED_phoH_13	Mediterranean surface water	AEQ27550	75/128	59	Group 6a
SH-phoH-85	128	GOM_phoH_09	Gulf of Mexico	AEQ27527	70/127	55	Group β
SH-phoH-86	129	MED_phoH_13	Mediterranean surface water	AEQ27550	75/128	59	Group 6a
SH-phoH-87	129	MED_phoH_13	Mediterranean surface water	AEQ27550	76/128	59	Group 6a
SH-phoH-88	129	MED_phoH_13	Mediterranean surface water	AEQ27550	76/128	59	Group 6a
SH-phoH-89	129	MED_phoH_13	Mediterranean surface water	AEQ27550	76/128	59	Group 6a
SH-phoH-90	129	MED_phoH_13	Mediterranean surface water	AEQ27550	76/128	59	Group 6a
SH-phoH-91	129	MED_phoH_13	Mediterranean surface water	AEQ27550	75/128	59	Group 6a
SH-phoH-92	129	MED_phoH_13	Mediterranean surface water	AEQ27550	76/128	59	Group 6a
SH-phoH-93	129	MED_phoH_13	Mediterranean surface water	AEQ27550	76/128	59	Group 6a
SH-phoH-94	129	MED_phoH_13	Mediterranean surface water	AEQ27550	76/128	59	Group 6a
SH-phoH-95	129	MED_phoH_13	Mediterranean surface water	AEQ27550	76/128	59	Group 6a
SH-phoH-96	129	MED_phoH_13	Mediterranean surface water	AEQ27550	75/128	59	Group 6a
SH-phoH-97	128	GOM_phoH_09	Gulf of Mexico	AEQ27527	70/127	55	Group β
MDJ-phoH-1	128	GOM_phoH_09	Gulf of Mexico	AEQ27527	70/127	55	Group β
MDJ-phoH-2	128	GOM_phoH_09	Gulf of Mexico	AEQ27527	70/127	55	Group β
MDJ-phoH-3	128	GOM_phoH_09	Gulf of Mexico	AEQ27527	70/127	55	Group β
MDJ-phoH-4	128	GOM_phoH_09	Gulf of Mexico	AEQ27527	70/127	55	Group β
MDJ-phoH-5	128	GOM_phoH_09	Gulf of Mexico	AEQ27527	70/127	55	Group β
MDJ-phoH-6	128	SAR_0m_phoH_30	Sargasso Sea	AEQ27325	75/128	59	Group β
MDJ-phoH-7	128	GOM_phoH_09	Gulf of Mexico	AEQ27527	70/127	55	Group β
MDJ-phoH-8	128	GOM_phoH_09	Gulf of Mexico	AEQ27527	70/127	55	Group β
MDJ-phoH-9	128	GOM_phoH_09	Gulf of Mexico	AEQ27527	70/127	55	Group β
MDJ-phoH-10	128	SAR_0m_phoH_30	Sargasso Sea	AEQ27325	73/128	57	Group β
MDJ-phoH-11	128	GOM_phoH_09	Gulf of Mexico	AEQ27527	70/127	55	Group β
MDJ-phoH-12	128	GOM_phoH_09	Gulf of Mexico	AEQ27527	70/127	55	Group β
MDJ-phoH-13	128	SAR_phoH_Mar_2011_0m_04	Sargasso Sea	AKV56976	69/127	54	Group β
MDJ-phoH-14	128	GOM_phoH_09	Gulf of Mexico	AEQ27527	70/127	55	Group β
MDJ-phoH-15	128	GOM_phoH_09	Gulf of Mexico	AEQ27527	70/127	55	Group β
MDJ-phoH-16	128	GOM_phoH_09	Gulf of Mexico	AEQ27527	70/127	55	Group β
MDJ-phoH-17	128	GOM_phoH_09	Gulf of Mexico	AEQ27527	69/127	54	Group β
MDJ-phoH-18	128	SAR_0m_phoH_37	Sargasso Sea	AEQ27332	70/127	55	Group β
MDJ-phoH-19	128	SAR_phoH_Mar_2011_0m_04	Sargasso Sea	AKV56976	69/127	54	Group β
MDJ-phoH-20	128	GOM_phoH_09	Gulf of Mexico	AEQ27527	71/127	56	Group β
MDJ-phoH-21	128	GOM_phoH_09	Gulf of Mexico	AEQ27527	70/127	55	Group β
MDJ-phoH-22	128	GOM_phoH_09	Gulf of Mexico	AEQ27527	70/127	55	Group β
MDJ-phoH-23	128	GOM_phoH_09	Gulf of Mexico	AEQ27527	70/127	55	Group β
MDJ-phoH-24	128	GOM_phoH_09	Gulf of Mexico	AEQ27527	70/127	55	Group β
MDJ-phoH-25	128	GOM_phoH_09	Gulf of Mexico	AEQ27527	69/127	54	Group β
MDJ-phoH-26	128	GOM_phoH_09	Gulf of Mexico	AEQ27527	70/127	55	Group β
MDJ-phoH-27	128	GOM_phoH_09	Gulf of Mexico	AEQ27527	70/127	55	Group β
MDJ-phoH-28	128	SAR_phoH_Mar_2011_0m_04	Sargasso Sea	AKV56976	69/127	54	Group β
MDJ-phoH-29	128	GOM_phoH_09	Gulf of Mexico	AEQ27527	70/127	55	Group β
MDJ-phoH-30	128	GOM_phoH_09	Gulf of Mexico	AEQ27527	69/127	54	Group β
MDJ-phoH-31	128	GOM_phoH_09	Gulf of Mexico	AEQ27527	69/127	54	Group β
MDJ-phoH-32	128	GOM_phoH_09	Gulf of Mexico	AEQ27527	70/127	55	Group β
MDJ-phoH-33	128	GOM_phoH_09	Gulf of Mexico	AEQ27527	70/127	55	Group β
MDJ-phoH-34	128	GOM_phoH_09	Gulf of Mexico	AEQ27527	68/127	54	Group β
MDJ-phoH-35	128	GOM_phoH_09	Gulf of Mexico	AEQ27527	70/127	55	Group β
MDJ-phoH-36	128	GOM_phoH_09	Gulf of Mexico	AEQ27527	70/127	55	Group β
DA-phoH-1	135	*Synechococcus* phage S-SSM7	Sargasso Sea	YP_004324370	115/134	86	Group 2a
DA-phoH-2	135	uncultured Mediterranean phage uvMED	Mediterranean Sea	BAR30472	105/134	78	Group 2b
DA-phoH-3	135	*Synechococcus* phage S-SSM7	Sargasso Sea	YP_004324370	114/134	85	Group 2a
DA-phoH-4	135	*Synechococcus* phage S-SSM7	Sargasso Sea	YP_004324370	115/134	86	Group 2a
DA-phoH-5	135	*Synechococcus* phage S-SSM7	Sargasso Sea	YP_004324370	115/134	86	Group 2a
DA-phoH-6	135	*Synechococcus* phage S-SSM7	Sargasso Sea	YP_004324370	113/134	84	Group 2a
DA-phoH-7	135	uncultured Mediterranean phage uvMED	Mediterranean Sea	BAR30472	106/134	79	Group 2b
DA-phoH-8	135	*Synechococcus* phage S-SSM7	Sargasso Sea	YP_004324370	115/134	86	Group 2a
DA-phoH-9	129	MED_phoH_02	Mediterranean Sea	AEQ27539	79/128	62	Group 6c
DA-phoH-10	135	uncultured Mediterranean phage uvMED	Mediterranean Sea	BAR30472	107/134	80	Group 2b
DA-phoH-11	135	uncultured Mediterranean phage uvMED	Mediterranean Sea	BAR30472	106/134	79	Group 2b
DA-phoH-12	135	uncultured Mediterranean phage uvMED	Mediterranean Sea	BAR30472	105/134	78	Group 2b
DA-phoH-13	135	*Synechococcus* phage S-SSM7	Sargasso Sea	YP_004324370	115/134	86	Group 2a
DA-phoH-14	135	*Synechococcus* phage S-SSM7	Sargasso Sea	YP_004324370	115/134	86	Group 2a
DA-phoH-15	135	*Synechococcus* phage S-RSM4	Red Sea	YP_003097238	114/134	85	Group 2a
DA-phoH-16	129	SAR_0m_phoH_30	Sargasso Sea	AEQ27325	66/128	52	Group α
DA-phoH-17	135	*Synechococcus* phage S-SSM7	Sargasso Sea	YP_004324370	115/134	86	Group 2a
DA-phoH-18	135	uncultured Mediterranean phage uvMED	Mediterranean Sea	BAR30472	122/134	91	Group 2a
DA-phoH-19	129	MED_phoH_02	Mediterranean Sea	AEQ27539	77/128	60	Group 6c
DA-phoH-20	135	*Synechococcus* phage S-SSM7	Sargasso Sea	YP_004324370	115/134	86	Group 2a
DA-phoH-21	129	MED_phoH_02	Mediterranean Sea	AEQ27539	77/128	60	Group 6c
DA-phoH-22	135	*Synechococcus* phage S-SSM7	Sargasso Sea	YP_004324370	115/134	86	Group 2a
DA-phoH-23	135	*Synechococcus* phage S-RSM4	Red Sea	YP_003097238	114/134	85	Group 2a
DA-phoH-24	135	uncultured Mediterranean phage uvMED	Mediterranean Sea	BAR30472	107/134	80	Group 2b
DA-phoH-25	129	MED_phoH_08	Mediterranean Sea	AEQ27545	77/128	60	Group 6c
DA-phoH-26	135	*Synechococcus* phage S-SSM7	Sargasso Sea	YP_004324370	121/134	90	Group 2a
DA-phoH-27	135	uncultured Mediterranean phage uvMED	Mediterranean Sea	BAR30472	106/134	79	Group 2b

DA-phoH-28	135	uncultured Mediterranean phage uvMED	Mediterranean Sea	BAR30472	120/134	90	Group 2a
DA-phoH-29	129	SAR_0m_phoH_30	Sargasso Sea	AEQ27325	77/128	60	Group 6c
DA-phoH-30	135	*Synechococcus* phage S-RSM4	Red Sea	YP_003097238	114/134	85	Group 2a
DA-phoH-31	135	uncultured Mediterranean phage uvMED	Mediterranean Sea	BAR30472	103/134	77	Group 2b
DA-phoH-32	135	*Synechococcus* phage S-SSM7	Sargasso Sea	YP_004324370	113/134	84	Group 2a
DA-phoH-33	135	*Synechococcus* phage S-SSM7	Sargasso Sea	YP_004324370	115/134	86	Group 2a
DA-phoH-34	129	MED_phoH_13	Mediterranean Sea	AEQ27550	77/128	60	Group 6a
DA-phoH-35	135	uncultured Mediterranean phage uvMED	Mediterranean Sea	BAR30472	106/134	79	Group 2b
DA-phoH-36	135	*Synechococcus* phage S-RSM4	Red Sea	YP_003097238	114/134	85	Group 2a
DA-phoH-37	135	*Synechococcus* phage S-SSM7	Sargasso Sea	YP_004324370	116/134	87	Group 2a
DA-phoH-38	135	*Synechococcus* phage S-RSM4	Red Sea	YP_003097238	114/134	85	Group 2a
DA-phoH-39	135	uncultured Mediterranean phage uvMED	Mediterranean Sea	BAR30472	107/134	80	Group 2b
DA-phoH-40	129	SAR_0m_phoH_30	Sargasso Sea	AEQ27325	66/128	52	Group α
DA-phoH-41	129	MED_phoH_02	Mediterranean Sea	AEQ27539	78/128	61	Group 6c
DA-phoH-42	135	*Synechococcus* phage S-SSM7	Sargasso Sea	YP_004324370	114/134	85	Group 2a
DA-phoH-43	135	*Synechococcus* phage S-SSM7	Sargasso Sea	YP_004324370	115/134	86	Group 2a
DA-phoH-44	135	uncultured Mediterranean phage uvMED	Mediterranean Sea	BAR30472	121/134	90	Group 2b
DA-phoH-45	135	*Synechococcus* phage S-SSM7	Sargasso Sea	YP_004324370	118/134	88	Group 2a
DA-phoH-46	135	uncultured Mediterranean phage uvMED	Mediterranean Sea	BAR30472	107/134	80	Group 2b
DA-phoH-47	135	*Synechococcus* phage S-SSM7	Sargasso Sea	YP_004324370	115/134	86	Group 2a
DA-phoH-48	129	MED_phoH_02	Mediterranean Sea	AEQ27539	78/128	61	Group 6c
DA-phoH-49	135	uncultured Mediterranean phage uvMED	Mediterranean Sea	BAR30472	106/134	79	Group 2b
DA-phoH-50	135	*Synechococcus* phage S-RSM4	Red Sea	YP_003097238	114/134	85	Group 2a
DA-phoH-51	135	*Synechococcus* phage S-RSM4	Red Sea	YP_003097238	113/134	84	Group 2a
DA-phoH-52	135	*Synechococcus* phage S-SSM7	Sargasso Sea	YP_004324370	115/134	86	Group 2a
DA-phoH-53	129	MED_phoH_02	Mediterranean Sea	AEQ27539	78/128	61	Group 6c
DA-phoH-54	129	MED_phoH_02	Mediterranean Sea	AEQ27539	78/128	61	Group 6c
DA-phoH-55	129	MED_phoH_02	Mediterranean Sea;	AEQ27539	78/128	61	Group 6c
DA-phoH-56	129	SAR_0m_phoH_30	Sargasso Sea	AEQ27325	65/128	51	Group α
DA-phoH-57	135	*Synechococcus* phage S-RSM4	Red Sea	YP_003097238	115/134	86	Group 2a
DA-phoH-58	135	*Synechococcus* phage S-SSM7	Sargasso Sea	YP_004324370	115/134	86	Group 2a
DA-phoH-59	135	*Synechococcus* phage S-SSM7	Sargasso Sea	YP_004324370	114/134	85	Group 2a
DA-phoH-60	135	*Synechococcus* phage S-SSM7	Sargasso Sea	YP_004324370	115/134	86	Group 2a
DA-phoH-61	129	MED_phoH_02	Mediterranean Sea	AEQ27539	78/128	61	Group 6c
DA-phoH-62	135	*Synechococcus* phage S-RSM4	Red Sea	YP_003097238	114/134	85	Group 2a
DA-phoH-63	129	MED_phoH_02	Mediterranean Sea	AEQ27539	78/128	61	Group 6c
DA-phoH-64	129	MED_phoH_02	Mediterranean Sea	AEQ27539	78/128	61	Group 6c
DA-phoH-65	129	MED_phoH_02	Mediterranean Sea	AEQ27539	78/128	61	Group 6c
DA-phoH-66	135	*Synechococcus* phage S-RSM4	Red Sea	YP_003097238	114/134	85	Group 2a
DA-phoH-67	128	SAR_0m_phoH_30	Sargasso Sea	AEQ27325	81/128	63	Group 6d
DA-phoH-68	135	uncultured Mediterranean phage uvMED	Mediterranean Sea	BAR30472	119/134	89	Group 2a
DA-phoH-69	135	*Synechococcus* phage S-SSM7	Sargasso Sea	YP_004324370	115/134	86	Group 2a
DA-phoH-70	135	*Synechococcus* phage S-SSM7	Sargasso Sea	YP_004324370	114/134	85	Group 2a
DA-phoH-71	135	*Synechococcus* phage S-SSM7	Sargasso Sea	YP_004324370	113/134	84	Group 2a
DA-phoH-72	135	uncultured Mediterranean phage uvMED	Mediterranean Sea	BAR30472	123/134	92	Group 2a
DA-phoH-73	135	*Synechococcus* phage S-SSM7	Sargasso Sea	YP_004324370	115/134	86	Group 2a
DA-phoH-74	135	*Synechococcus* phage S-RSM4	Red Sea	YP_003097238	113/134	84	Group 2a
DA-phoH-75	135	uncultured Mediterranean phage uvMED	Mediterranean Sea	BAR30472	105/134	78	Group 2b
DA-phoH-76	135	uncultured Mediterranean phage uvMED	Mediterranean Sea	BAR30472	104/134	78	Group 2b
DA-phoH-77	128	SAR_0m_phoH_30	Sargasso Sea	AEQ27325	76/128	59	Group β
DA-phoH-78	135	*Synechococcus* phage S-SSM7	Sargasso Sea	YP_004324370	115/134	86	Group 2a
DA-phoH-79	135	*Synechococcus* phage S-SSM7	Sargasso Sea	YP_004324370	115/134	86	Group 2a
DA-phoH-80	135	uncultured Mediterranean phage uvMED	Mediterranean Sea	BAR30472	106/134	79	Group 2b
DA-phoH-81	135	*Synechococcus* phage S-SSM7	Sargasso Sea	YP_004324370	115/134	86	Group 2a
DA-phoH-82	135	*Synechococcus* phage S-SSM7	Sargasso Sea	YP_004324370	114/134	85	Group 2a
DA-phoH-83	135	*Synechococcus* phage S-SSM7	Sargasso Sea	YP_004324370	114/134	85	Group 2a
DA-phoH-84	135	*Synechococcus* phage S-SSM7	Sargasso Sea	YP_004324370	115/134	86	Group 2a
DA-phoH-85	129	MED_phoH_02	Mediterranean Sea	AEQ27539	78/128	61	Group 6c
DA-phoH-86	135	*Synechococcus* phage S-SSM7	Sargasso Sea	YP_004324370	114/134	85	Group 2a
DA-phoH-87	135	*Synechococcus* phage S-SSM7	Sargasso Sea	YP_004324370	116/134	87	Group 2a
DA-phoH-88	129	MED_phoH_02	Mediterranean Sea	AEQ27539	77/128	60	Group 6c
DA-phoH-89	135	*Synechococcus* phage S-SSM7	Sargasso Sea	YP_004324370	120/134	90	Group 2a
DA-phoH-90	135	uncultured Mediterranean phage uvMED	Mediterranean Sea	BAR30472	107/134	80	Group 2b
DA-phoH-91	135	*Synechococcus* phage S-SSM7	Sargasso Sea	YP_004324370	114/134	85	Group 2a
DA-phoH-92	135	*Synechococcus* phage S-SSM7	Sargasso Sea	YP_004324370	115/134	86	Group 2a
DA-phoH-93	129	MED_phoH_02	Mediterranean Sea	AEQ27539	78/128	61	Group 6c
DA-phoH-94	135	*Synechococcus* phage S-RSM4	Red Sea	YP_003097238	114/134	85	Group 2a
DA-phoH-95	129	MED_phoH_08	Mediterranean Sea	AEQ27545	77/128	60	Group 6c
DA-phoH-96	129	MED_phoH_13	Mediterranean Sea	AEQ27550	77/128	60	Group 6a
DA-phoH-97	129	SAR_0m_phoH_30	Sargasso Sea	AEQ27325	65/128	51	Group α
DA-phoH-98	135	*Synechococcus* phage S-SSM7	Sargasso Sea	YP_004324370	115/134	86	Group 2a
DA-phoH-99	129	MED_phoH_02	Mediterranean Sea	AEQ27539	78/128	61	Group 6c
DA-phoH-100	135	*Synechococcus* phage S-RSM4	Red Sea	YP_003097238	114/134	85	Group 2a
DA-phoH-101	135	*Synechococcus* phage S-SSM7	Sargasso Sea	YP_004324370	119/134	89	Group 4b
DA-phoH-102	135	uncultured Mediterranean phage uvMED	Mediterranean Sea	BAR30472	107/134	80	Group 2b
DA-phoH-103	135	*Synechococcus* phage S-SSM7	Sargasso Sea	YP_004324370	113/134	84	Group 2a
DA-phoH-104	129	SAR_0m_phoH_30	Sargasso Sea	AEQ27325	66/128	52	Group α
DA-phoH-105	135	uncultured Mediterranean phage uvMED	Mediterranean Sea	BAR30472	106/134	79	Group 2b
DA-phoH-106	135	*Synechococcus* phage S-SSM7	Sargasso Sea	YP_004324370	114/134	85	Group 2a
DA-phoH-107	135	*Synechococcus* phage S-SSM7	Sargasso Sea	YP_004324370	115/134	86	Group 2a
DA-phoH-108	135	*Synechococcus* phage S-SSM7	Sargasso Sea	YP_004324370	115/134	86	Group 2a
DA-phoH-109	135	*Synechococcus* phage S-SSM7	Sargasso Sea	YP_004324370	115/134	86	Group 2a
DA-phoH-110	129	MED_phoH_02	Mediterranean Sea	AEQ27539	78/128	61	Group 6c
DA-phoH-111	135	*Synechococcus* phage S-RSM4	Red Sea	YP_003097238	114/134	85	Group 2a

DA-phoH-112	135	uncultured Mediterranean phage uvMED	Mediterranean Sea	BAR30472	107/134	80	Group 2b
DA-phoH-113	135	*Synechococcus* phage S-SSM7	Sargasso Sea	YP_004324370	119/134	89	Group 2a
DA-phoH-114	129	MED_phoH_13	Mediterranean Sea	AEQ27550	76/128	59	Group 6e
DA-phoH-115	135	*Synechococcus* phage S-SSM7	Sargasso Sea	YP_004324370	115/134	86	Group 2a
DA-phoH-116	135	*Synechococcus* phage S-SSM7	Sargasso Sea	YP_004324370	115/134	86	Group 2a
DA-phoH-117	135	*Synechococcus* phage S-SSM7	Sargasso Sea	YP_004324370	114/134	85	Group 2a
DA-phoH-118	135	*Synechococcus* phage S-SSM7	Sargasso Sea	YP_004324370	114/134	85	Group 2a
DA-phoH-119	129	MED_phoH_02	Mediterranean Sea	AEQ27539	77/128	60	Group 6c
DA-phoH-120	135	*Synechococcus* phage S-SSM7	Sargasso Sea	YP_004324370	115/134	86	Group 2a
DA-phoH-121	135	*Synechococcus* phage S-SSM7	Sargasso Sea	YP_004324370	115/134	86	Group 2a
DA-phoH-122	135	uncultured Mediterranean phage uvMED	Mediterranean Sea	BAR30472	107/134	80	Group 2b
DA-phoH-123	135	*Synechococcus* phage S-SSM7	Sargasso Sea	YP_004324370	115/134	86	Group 2a
DA-phoH-124	129	SAR_0m_phoH_30	Sargasso Sea	AEQ27325	77/128	60	Group 6c
DA-phoH-125	135	*Synechococcus* phage S-RSM4	Red Sea	YP_003097238	114/134	85	Group 2a
DA-phoH-126	129	MED_phoH_02	Mediterranean Sea	AEQ27539	78/128	61	Group 6c
DA-phoH-127	135	*Synechococcus* phage S-SSM7	Sargasso Sea	YP_004324370	115/134	86	Group 2a
DA-phoH-128	129	MED_phoH_02	Mediterranean Sea	AEQ27539	78/128	61	Group 6c
DA-phoH-129	135	*Synechococcus* phage S-SSM7	Sargasso Sea	YP_004324370	115/134	86	Group 2a
DA-phoH-130	135	*Synechococcus* phage S-RSM4	Red Sea	YP_003097238	113/134	84	Group 2a
DA-phoH-131	129	MED_phoH_02	Mediterranean Sea	AEQ27539	78/128	61	Group 6c
DA-phoH-132	135	uncultured Mediterranean phage uvMED	Mediterranean Sea	BAR30472	107/134	80	Group 2b
DA-phoH-133	135	*Synechococcus* phage S-RSM4	Red Sea	YP_003097238	114/134	85	Group 2a
DA-phoH-134	135	*Synechococcus* phage S-SSM7	Sargasso Sea	YP_004324370	115/134	86	Group 2a
DA-phoH-135	135	*Synechococcus* phage S-SSM7	Sargasso Sea	YP_004324370	115/134	86	Group 2a
DA-phoH-136	129	SAR_0m_phoH_30	Sargasso Sea	AEQ27325	66/128	52	Group α
DA-phoH-137	135	uncultured Mediterranean phage uvMED	Mediterranean Sea	BAR30472	107/134	80	Group 2b
DA-phoH-138	135	*Synechococcus* phage S-SSM7	Sargasso Sea	YP_004324370	115/134	86	Group 2a
DA-phoH-139	135	*Synechococcus* phage S-SSM7	Sargasso Sea	YP_004324370	115/134	86	Group 2a
DA-phoH-140	135	*Synechococcus* phage S-SSM7	Sargasso Sea	YP_004324370	115/134	86	Group 2a
DA-phoH-141	135	*Synechococcus* phage S-SSM7	Sargasso Sea	YP_004324370	115/134	86	Group 2a
DA-phoH-142	135	uncultured Mediterranean phage uvMED	Mediterranean Sea	BAR30472	107/134	80	Group 2b
DA-phoH-143	129	MED_phoH_02	Mediterranean Sea;	AEQ27539	78/128	61	Group 6c
DA-phoH-144	135	*Synechococcus* phage S-SSM7	Sargasso Sea	YP_004324370	118/134	88	Group 2a
DA-phoH-145	135	*Synechococcus* phage S-SSM7	Sargasso Sea	YP_004324370	115/134	86	Group 2a
DA-phoH-146	135	*Synechococcus* phage S-SSM7	Sargasso Sea	YP_004324370	115/134	86	Group 2a
DA-phoH-147	135	*Synechococcus* phage S-SSM7	Sargasso Sea	YP_004324370	115/134	86	Group 2a
DA-phoH-148	135	*Synechococcus* phage S-SSM7	Sargasso Sea	YP_004324370	115/134	86	Group 2a
DA-phoH-149	135	*Synechococcus* phage S-SSM7	Sargasso Sea	YP_004324370	115/134	86	Group 2a
DA-phoH-150	135	*Synechococcus* phage S-SSM7	Sargasso Sea	YP_004324370	115/134	86	Group 2a
DA-phoH-151	135	*Synechococcus* phage S-SSM7	Sargasso Sea	YP_004324370	118/134	88	Group 2a
DA-phoH-152	135	uncultured Mediterranean phage uvMED	Mediterranean Sea	BAR30472	121/134	90	Group 2b
DA-phoH-153	135	uncultured Mediterranean phage uvMED	Mediterranean Sea	BAR30472	106/134	79	Group 2b
DA-phoH-154	135	*Synechococcus* phage S-SSM7	Sargasso Sea	YP_004324370	114/134	85	Group 2a
DA-phoH-155	135	*Synechococcus* phage S-SSM7	Sargasso Sea	YP_004324370	114/134	85	Group 2a
DA-phoH-156	135	*Synechococcus* phage S-SSM7	Sargasso Sea	YP_004324370	116/134	87	Group 2a
DA-phoH-157	135	*Synechococcus* phage S-SSM7	Sargasso Sea	YP_004324370	115/134	86	Group 2a
DA-phoH-158	135	*Synechococcus* phage S-RSM4	Red Sea	YP_003097238	114/134	85	Group 2a
DA-phoH-159	129	MED_phoH_02	Mediterranean Sea	AEQ27539	78/128	61	Group 6c
DA-phoH-160	135	*Synechococcus* phage S-SSM7	Sargasso Sea	YP_004324370	115/134	86	Group 2a
DA-phoH-161	135	*Synechococcus* phage S-RSM4	Red Sea	YP_003097238	114/134	85	Group 2a
DA-phoH-162	129	MED_phoH_02	Mediterranean Sea	AEQ27539	78/128	61	Group 6c
DA-phoH-163	135	*Synechococcus* phage S-RSM4	Red Sea	YP_003097238	113/134	84	Group 2a
DA-phoH-164	135	*Synechococcus* phage S-SSM7	Sargasso Sea	YP_004324370	115/134	86	Group 2a
DA-phoH-165	135	uncultured Mediterranean phage uvMED	Mediterranean Sea	BAR30472	107/134	80	Group 2b
DA-phoH-166	129	SAR_1000m_phoH_14	Sargasso Sea	AEQ27392	77/126	61	Group 2a
YJG-phoH-1	126	SAR_1000m_phoH_14	Sargasso Sea	AEQ27392	84/125	67	Group 2a
YJG-phoH-2	126	SAR_1000m_phoH_16	Sargasso Sea	AEQ27394	87/125	70	Group 2a
YJG-phoH-3	126	SAR_1000m_phoH_14	Sargasso Sea	AEQ27392	84/125	67	Group 2a
YJG-phoH-4	126	SAR_1000m_phoH_16	Sargasso Sea	AEQ27394	87/125	70	Group 2a
YJG-phoH-5	126	SAR_1000m_phoH_16	Sargasso Sea	AEQ27394	87/125	70	Group 6b
YJG-phoH-6	126	SAR_1000m_phoH_14	Sargasso Sea	AEQ27392	85/125	68	Group 6b
YJG-phoH-7	127	SAR_1000m_phoH_14	Sargasso Sea	AEQ27392	61/126	48	Group 2a
YJG-phoH-8	126	SAR_1000m_phoH_16	Sargasso Sea	AEQ27394	85/125	68	Group 2a
YJG-phoH-9	126	SAR_1000m_phoH_14	Sargasso Sea	AEQ27392	85/125	68	Group 2a
YJG-phoH-10	126	SAR_1000m_phoH_16	Sargasso Sea	AEQ27394	86/125	69	Group 6b
YJG-phoH-11	126	SAR_1000m_phoH_16	Sargasso Sea	AEQ27394	87/125	70	Group 2a
YJG-phoH-12	127	SAR_1000m_phoH_14	Sargasso Sea	AEQ27392	61/126	48	Group 2a
YJG-phoH-13	127	*Acinetobacter* phage YMC13/03/R2096	South Korea	YP_009146825	58/124	47	Group 2a
YJG-phoH-14	127	*Acinetobacter* phage YMC13/03/R2096	South Korea	YP_009146825	57/124	46	Group 2a
YJG-phoH-15	127	*Acinetobacter* phage YMC13/03/R2096	South Korea	YP_009146825	57/124	46	Group 2a
YJG-phoH-16	127	*Acinetobacter* phage YMC13/03/R2096	South Korea	YP_009146825	58/124	47	Group 2a
YJG-phoH-17	127	*Acinetobacter* phage YMC13/03/R2096	South Korea	YP_009146825	58/124	47	Group 6a
YJG-phoH-18	127	*Acinetobacter* phage YMC13/03/R2096	South Korea	YP_009146825	58/124	47	Group 2a
YJG-phoH-19	127	*Acinetobacter* phage YMC13/03/R2096	South Korea	YP_009146825	57/124	46	Group β
YJG-phoH-20	127	*Acinetobacter* phage YMC13/03/R2096	South Korea	YP_009146825	57/124	46	Group 6b
YJG-phoH-21	127	*Acinetobacter* phage YMC13/03/R2096	South Korea	YP_009146825	58/124	47	Group 4b
YJG-phoH-22	127	*Acinetobacter* phage YMC13/03/R2096	South Korea	YP_009146825	58/124	47	Group 4b
YJG-phoH-23	127	*Acinetobacter* phage YMC13/03/R2096	South Korea	YP_009146825	58/124	47	Group 4b
YJG-phoH-24	127	*Acinetobacter* phage YMC13/03/R2096	South Korea	YP_009146825	56/124	45	Group 2a
YJG-phoH-25	127	*Acinetobacter* phage YMC13/03/R2096	South Korea	YP_009146825	58/124	47	Group 6b
YJG-phoH-26	127	*Acinetobacter* phage YMC13/03/R2096	South Korea	YP_009146825	58/124	47	Group 2a
YJG-phoH-27	127	*Acinetobacter* phage YMC13/03/R2096	South Korea	YP_009146825	57/124	46	Group 2a
YJG-phoH-28	127	*Acinetobacter* phage YMC13/03/R2096	South Korea	YP_009146825	58/124	47	Group 2a
YJG-phoH-29	127	*Acinetobacter* phage YMC13/03/R2096	South Korea	YP_009146825	56/124	45	Group 3e

YJG-phoH-30	127	*Acinetobacter* phage YMC13/03/R2096	South Korea	YP_009146825	58/124	47	Group 2a
YJG-phoH-31	127	*Acinetobacter* phage YMC13/03/R2096	South Korea	YP_009146825	57/124	46	Group 4b
YJG-phoH-32	127	*Acinetobacter* phage YMC13/03/R2096	South Korea	YP_009146825	58/124	47	Group 2a
YJG-phoH-33	127	*Acinetobacter* phage YMC13/03/R2096	South Korea	YP_009146825	58/124	47	Group 6b
YJG-phoH-34	127	*Acinetobacter* phage YMC13/03/R2096	South Korea	YP_009146825	58/124	47	Group 6b
YJG-phoH-35	127	*Acinetobacter* phage YMC13/03/R2096	South Korea	YP_009146825	58/124	47	Group 2a
YJG-phoH-36	127	*Acinetobacter* phage YMC13/03/R2096	South Korea	YP_009146825	58/124	47	Group 2a
YJG-phoH-37	127	*Acinetobacter* phage YMC13/03/R2096	South Korea	YP_009146825	58/124	47	Group 2a
YJG-phoH-38	127	*Acinetobacter* phage YMC13/03/R2096	South Korea	YP_009146825	58/124	47	Group 2a
YJG-phoH-39	127	*Acinetobacter* phage YMC13/03/R2096	South Korea	YP_009146825	58/124	47	Group 4b
YJG-phoH-40	127	*Acinetobacter* phage YMC13/03/R2096	South Korea	YP_009146825	58/124	47	Group 4b
YJG-phoH-41	127	*Acinetobacter* phage YMC13/03/R2096	South Korea	YP_009146825	58/124	47	Group 2a
YJG-phoH-42	127	*Acinetobacter* phage YMC13/03/R2096	South Korea	YP_009146825	58/124	47	Group 2a
YJG-phoH-43	127	*Acinetobacter* phage YMC13/03/R2096	South Korea	YP_009146825	58/124	47	Group 6b
YJG-phoH-44	127	*Acinetobacter* phage YMC13/03/R2096	South Korea	YP_009146825	56/124	45	Group γ
YJG-phoH-45	128	SAR_phoH_27	Sargasso Sea	AEQ27433	85/128	66	Group 4b
YJG-phoH-46	128	SAR_phoH_27	Sargasso Sea	AEQ27433	83/128	65	Group 6b
YJG-phoH-47	128	SAR_phoH_27	Sargasso Sea	AEQ27433	85/128	66	Group 2a
YJG-phoH-48	128	SAR_phoH_27	Sargasso Sea	AEQ27433	85/128	66	Group 2a
YJG-phoH-49	129	MED_phoH_02	Mediterranean Sea	AEQ27539	80/128	63	Group 2a
YJG-phoH-50	129	SAR_phoH_67	Sargasso Sea	AEQ27473	81/128	63	Group 4b
YJG-phoH-51	128	SAR_phoH_27	Sargasso Sea	AEQ27433	85/128	66	Group 6b
YJG-phoH-52	128	SAR_phoH_27	Sargasso Sea	AEQ27433	85/128	66	Group 6b
YJG-phoH-53	128	SAR_phoH_27	Sargasso Sea	AEQ27433	85/128	66	Group 2a
YJG-phoH-54	129	MED_phoH_08	Mediterranean Sea	AEQ27545	80/128	63	Group 6b
YJG-phoH-55	128	SAR_phoH_27	Sargasso Sea	AEQ27433	85/128	66	Group 6a
YJG-phoH-56	128	SAR_phoH_27	Sargasso Sea	AEQ27433	85/128	66	Group 3e
YJG-phoH-57	128	SAR_100m_phoH_05	Sargasso Sea	AEQ27480	80/130	62	Group 2a
YJG-phoH-58	128	SAR_phoH_27	Sargasso Sea	AEQ27433	85/128	66	Group 2a
YJG-phoH-59	128	SAR_phoH_27	Sargasso Sea	AEQ27433	83/128	65	Group 6b
YJG-phoH-60	128	SAR_phoH_27	Sargasso Sea	AEQ27433	84/128	66	Group 2a
YJG-phoH-61	129	MED_phoH_02	Mediterranean Sea	AEQ27539	80/128	63	Group 6b
YJG-phoH-62	198	SAR_phoH_27	Sargasso Sea	AEQ27433	84/128	66	Group 4b
YJG-phoH-63	129	SAR_phoH_27	Sargasso Sea	AEQ27433	77/128	60	Group 2a
YJG-phoH-64	129	SAR_phoH_67	Sargasso Sea	AEQ27473	81/128	63	Group 3e
YJG-phoH-65	128	SAR_phoH_27	Sargasso Sea	AEQ27433	85/128	66	Group 2a
YJG-phoH-66	129	MED_phoH_08	Mediterranean Sea	AEQ27545	79/128	62	Group 2a
YJG-phoH-67	128	SAR_phoH_27	Sargasso Sea	AEQ27433	84/128	66	Group 2a
YJG-phoH-68	128	SAR_phoH_27	Sargasso Sea	AEQ27433	85/128	66	Group 6b
YJG-phoH-69	128	SAR_phoH_27	Sargasso Sea	AEQ27433	85/128	66	Group 6b
YJG-phoH-70	129	SAR_0m_phoH_30	Sargasso Sea	AEQ27325	73/128	57	Group 2a
YJG-phoH-71	128	SAR_phoH_27	Sargasso Sea	AEQ27433	83/128	65	Group 4b
YJG-phoH-72	128	SAR_phoH_27	Sargasso Sea	AEQ27433	83/128	65	Group 2a
YJG-phoH-73	129	MED_phoH_02	Mediterranean Sea	AEQ27539	79/128	62	Group δ
YJG-phoH-74	129	SAR_phoH_67	Sargasso Sea	AEQ27473	80/128	63	Group δ
YJG-phoH-75	128	SAR_100m_phoH_05	Sargasso Sea	AEQ27480	80/130	62	Group δ
YJG-phoH-76	128	SAR_100m_phoH_05	Sargasso Sea	AEQ27480	80/130	62	Group δ
YJG-phoH-77	128	SAR_100m_phoH_05	Sargasso Sea	AEQ27480	80/130	62	Group δ
YJG-phoH-78	128	SAR_phoH_27	Sargasso Sea	AEQ27433	85/128	66	Group δ
YJG-phoH-79	128	SAR_phoH_27	Sargasso Sea	AEQ27433	84/128	66	Group δ
YJG-phoH-80	128	SAR_phoH_27	Sargasso Sea	AEQ27433	84/128	66	Group δ
YJG-phoH-81	129	SAR_phoH_67	Sargasso Sea	AEQ27473	81/128	63	Group δ
YJG-phoH-82	129	SAR_phoH_67	Sargasso Sea	AEQ27473	81/128	63	Group δ
YJG-phoH-83	128	SAR_phoH_27	Sargasso Sea	AEQ27433	84/128	66	Group δ
YJG-phoH-84	128	SAR_phoH_27	Sargasso Sea	AEQ27433	85/128	66	Group δ
YJG-phoH-85	129	SAR_phoH_27	Sargasso Sea	AEQ27433	85/128	66	Group δ
YJG-phoH-86	129	SAR_phoH_67	Sargasso Sea	AEQ27473	80/128	63	Group δ
YJG-phoH-87	129	MED_phoH_08	Mediterranean Sea	AEQ27545	80/128	63	Group δ
YJG-phoH-88	128	SAR_100m_phoH_05	Sargasso Sea	AEQ27480	80/130	62	Group δ
YJG-phoH-89	129	MED_phoH_02	Mediterranean Sea	AEQ27539	80/128	63	Group δ
YJG-phoH-90	129	MED_phoH_08	Mediterranean Sea	AEQ27545	77/128	60	Group δ
YJG-phoH-91	129	BBC_phoH_01	British Columbia	AEQ27556	74/128	58	Group δ
YJG-phoH-92	128	SAR_100m_phoH_05	Sargasso Sea	AEQ27480	81/130	62	Group δ
YJG-phoH-93	128	SAR_phoH_27	Sargasso Sea	AEQ27433	84/128	66	Group δ
YJG-phoH-94	129	MED_phoH_08	Mediterranean Sea;	AEQ27545	80/128	63	Group δ
YJG-phoH-95	128	SAR_0m_phoH_37	Sargasso Sea	AEQ27332	80/127	63	Group δ
YJG-phoH-96	129	MED_phoH_08	Mediterranean Sea;	AEQ27545	80/128	63	Group δ
YJG-phoH-97	128	SAR_phoH_27	Sargasso Sea	AEQ27433	84/128	66	Group δ
YJG-phoH-98	129	BBC_phoH_01	British Columbia	AEQ27556	75/128	59	Group δ
YJG-phoH-99	128	SAR_phoH_27	Sargasso Sea	AEQ27433	84/128	66	Group δ
YJG-phoH-100	128	SAR_phoH_27	Sargasso Sea	AEQ27433	82/128	64	Group δ
YJG-phoH-101	128	SAR_100m_phoH_05	Sargasso Sea	AEQ27480	80/130	62	Group δ
YJG-phoH-102	129	MED_phoH_02	Mediterranean Sea	AEQ27539	78/128	61	Group δ
YJG-phoH-103	129	SAR_phoH_67	Sargasso Sea	AEQ27473	78/128	61	Group δ
YJG-phoH-104	128	SAR_phoH_27	Sargasso Sea	AEQ27433	85/128	66	Group δ
YJG-phoH-105	128	SAR_phoH_27	Sargasso Sea	AEQ27433	85/128	66	Group 2a
YJG-phoH-106	128	SAR_phoH_27	Sargasso Sea	AEQ27433	85/128	66	Group 6b
YJG-phoH-107	129	MED_phoH_08	Mediterranean Sea	AEQ27545	80/128	63	Group 2a
YJG-phoH-108	128	SAR_phoH_27	Sargasso Sea	AEQ27433	83/128	65	Group 2a
YJG-phoH-109	128	SAR_phoH_27	Sargasso Sea	AEQ27433	85/128	66	Group 6b
YJG-phoH-110	129	MED_phoH_02	Mediterranean Sea	AEQ27539	80/128	63	Group 3e
YJG-phoH-111	129	BBC_phoH_01	British Columbia	AEQ27556	74/128	58	Group 6b
YJG-phoH-112	129	MED_phoH_08	Mediterranean Sea	AEQ27545	77/128	60	Group 3e
YJG-phoH-113	129	BBC_phoH_01	British Columbia	AEQ27556	74/128	58	Group 6b

YJG-phoH-114	129	MED_phoH_08	Mediterranean Sea	AEQ27545	80/128	63	Group 6a
YJG-phoH-115	129	MED_phoH_08	Mediterranean Sea	AEQ27545	77/128	60	Group 2a
YJG-phoH-116	128	SAR_100m_phoH_05	Sargasso Sea	AEQ27480	81/130	62	Group 6b
YJG-phoH-117	129	MED_phoH_02	Mediterranean Sea	AEQ27539	78/128	61	Group 2a
YJG-phoH-118	128	SAR_phoH_27	Sargasso Sea	AEQ27433	85/128	66	Group 2a
YJG-phoH-119	128	SAR_100m_phoH_05	Sargasso Sea	AEQ27480	80/130	62	Group 2a
YJG-phoH-120	128	SAR_phoH_27	Sargasso Sea	AEQ27433	83/128	65	Group 2a
YJG-phoH-121	128	SAR_phoH_27	Sargasso Sea	AEQ27433	85/128	66	Group 6b
YJG-phoH-122	129	SAR_phoH_67	Sargasso Sea	AEQ27473	81/128	63	Group γ
YJG-phoH-123	129	SAR_phoH_67	Sargasso Sea	AEQ27473	81/128	63	Group 6b
YJG-phoH-124	128	SAR_phoH_27	Sargasso Sea	AEQ27433	84/128	66	Group 2a
YJG-phoH-125	129	MED_phoH_08	Mediterranean Sea	AEQ27545	80/128	63	Group 6b

[a]The length of amino acid residues.

[b]Accession number of amino acid sequences.

Table S4. The statistical overview of the closest relative of the *phoH* clones from the four paddy water samples in this study.

Source	Accession number	Clone/isolate	Daan (166)[a]		Suihua (97)[a]		Mudanjiang (36)[a]		Yanjiagang (125)[a]	
			Number of clones	Identity (%)	Number of clones	Identity (%)	Number of clones	Identity (%)	Number of clones	Identity (%)
Sargasso Sea	YP_004324370	*Synechococcus* phage S-SSM7	75	84-90						
	AEQ27325	SAR_0m_phoH_30	10	51-63	2	57, 58	2	57, 59	1	57
	AEQ27392	SAR_1000m_phoH_14	1	61					6	48-68
	AEQ27324	SAR_0m_phoH_29			8	55, 56				
	AEQ27389	SAR_1000m_phoH_11			4	54, 55				
	AKV56976	SAR_phoH_Mar_2011_0m_04			1	54	3	54		
	AKV56959	SAR_phoH_Sept_2010_0m_09			3	84, 85				
	AKV57024	SAR_phoH_Sept_2011_100m_02			2	55, 56				
	AEQ27332	SAR_0m_phoH_37					1	55	1	63
	AEQ27433	SAR_phoH_27							39	60-66
	AEQ27473	SAR_phoH_67							9	61, 63
	AEQ27480	SAR_100m_phoH_05							9	62
	AEQ27394	SAR_1000m_phoH_16							6	68-70
Mediterranean Sea	BAR30472	uncultured phage uvMED	31	77-92						
	AEQ27539	MED_phoH_02	24	60-62					7	61-63
	AEQ27545	MED_phoH_08	2	60					11	60-63
	AEQ27550	MED_phoH_13	3	59, 60	67	57-60				
Gulf of Mexico	AEQ27527	GOM_phoH_09			10	55	30	54-56		
British Columbia	AEQ27556	BBC_phoH_01							4	58, 59
Red Sea	YP_003097238	*Synechococcus* phage S-RSM4	20	84-86						
South Korea	YP_009146825	*Acinetobacter* phage YMC13/03/R2096							32	45-47

[a]Number in parenthesis is the total number of clones obtained from each sample.
